# 支气管镜介入技术治疗典型类癌的近期疗效及远期疗效观察

**DOI:** 10.3779/j.issn.1009-3419.2021.103.13

**Published:** 2021-12-20

**Authors:** 照华 夏, 书方 王, 芳 秦, 坤 乔, 云芝 周

**Affiliations:** 1 518000 深圳，深圳市第三人民医院胸外科 Department of Thoracic Surgery, The Third People's Hospital of Shenzhen, Shenzhen 518000, China; 2 100000 北京，北京应急总医院呼吸内科 Department of Respiratory Medicine, Emergency General Hospital, Beijing 100000, China

**Keywords:** 典型类癌, 支气管介入治疗, 疗效, Typical carcinoid, Interventional bronchoscopy, Efficacy

## Abstract

**背景与目的:**

支气管镜下的各种介入治疗技术包括圈套、二氧化碳冷冻、氩等离子体凝固（argon plasma coagulation, APC）、激光（Neodymium-dopted Yttrium Aluminium Garnet, Nd: YAG）、光动力治疗（photodynamic therapy, PDT），以上治疗技术通常用于治疗气管腔内肿瘤，但用于治疗典型类癌（typical carcinoid, TC）的研究并不普遍。本研究拟探讨支气管镜介入技术治疗典型类癌的近期疗效和远期疗效。

**方法:**

回顾性分析北京应急总医院2010年12月-2020年12月可消瘤的腔内典型类癌患者的临床资料，采用*Wilcoxon*秩和检验和卡方检验进行统计分析。

**结果:**

共纳入32例患者，术前支气管动脉栓塞18例（栓塞率56%，95%CI: 31%-79%）。呼吸困难分级评分治疗后较治疗前下降，差异有统计学意义[（1.44±1.03）分*vs*（0.25±0.58）分，*P*=0.003]；支气管管腔狭窄程度治疗后较治疗前改善，差异有统计学意义[(87.50%±13.90%) *vs* (17.50%±6.83%), *P* < 0.001]；支气管内径治疗后较治疗前扩宽，差异有统计学意义[(0.14±0.18) cm *vs* (0.84±0.29) cm, *P* < 0.001]。近期有效率为100%。32例患者中24例支气管镜介入治疗能够完全消瘤，完全缓解（complete remission, CR）率为75%。

**结论:**

支气管镜介入治疗技术治疗典型类癌的近期疗效及远期疗效显著。

## Abstract (en)

**Background and objective:**

The curative potential of various bronchoscopic treatments such as electric snare, carbon dioxide freezing, argon plasma coagulation (APC), Neudymium-dopted Yttrium Aluminium Garnet (Nd: YAG) laser and photodynamic therapy (PDT) for the treatment of intraluminal tumor has been administered previously, but this regimen is not common in the treatment of typical carcinoid. The aim of this study is to investigate the curative effects both in short-term and long-term of interventional bronchoscopy in the treatment of typical carcinoid.

**Methods:**

We retrospectively reviewed the clinical data of typical carcinoid patients who were treated with interventional bronchoscopy for tumor suppression and they were hospitalized in the Emergency General Hospital from December 2010 to December 2020, and *Wilcoxon* rank sum test and *chi-square* test were used for analysis.

**Results:**

A total of 32 patients were included, including 18 cases of preoperative bronchial artery embolization (embolization rate 56%, 95%CI: 31%-79%). The grade score of dyspnea decreased from before treatment to after treatment, and the difference was statistically significant [(1.44±1.03) score *vs* (0.25±0.58) score, *P*=0.003]; The degree of bronchial stenosis decreased from pre-treatment to post-treatment, and the difference was statistically significant [(87.50%±13.90%) *vs* (17.50%±6.83%), *P* < 0.001]; There was significant difference in bronchial diameter before and after treatment [(0.14±0.18) cm *vs* (0.84±0.29) cm, *P* < 0.001].

**Conclusion:**

Bronchoscopic interventional therapy has significant short-term and long-term effects in the treatment of typical carcinoid.

支气管类癌是肺部低度恶性的神经内分泌肿瘤，发病率占肺部肿瘤的1%-2%^[1]^。类癌根据不同的组织分化程度分为典型类癌（typical carcinoid, TC）和不典型类癌（atypical carcinoid, AC），其共同特征是肿瘤细胞可以分泌特定的肽类激素，如突触素（synaptophysin, Syn）、嗜铬素A（chromogranin A, CgA）等。

当前，支气管镜介入治疗技术一种或多种联合，能够治疗气管支气管腔内恶性肿瘤^[2]^。支气管腔内肿瘤往往影像学难以发现，支气管镜介入治疗尤其是光动力疗法（PDT），对早期浸润癌及低度恶性肿瘤可达到根治的效果，可能成为一种可替代手术的治疗方案^[3]^。外科手术是治疗支气管类癌的首选方法，并且大部分手术患者有很好的远期生存^[4]^，支气管镜介入治疗仅仅在有手术切除禁忌患者或拒绝手术患者中进行。手术前进行支气管镜介入治疗，可以更好地评估肿瘤生长情况和打通气道，阻塞性肺炎患者手术前还能够改善肺功能。

既往有学者^[5]^报道，TC患者行支气管镜介入治疗，介入治疗后实施外科手术，手术切除标本病理学未见肿瘤细胞。支气管镜介入治疗可能会提供一种最大程度保留肺功能的治疗策略。本研究拟探讨支气管镜介入治疗技术治疗典型类癌的近期疗效和远期疗效。

## 1 资料和方法

### 1.1 一般资料

回顾性分析北京应急总医院2010年12月-2020年12月收治支气管腔内典型类癌患者的临床资料，纳入标准：①组织学分型为典型类癌；②使用高分辨率计算机体层X线摄影术（high-resolution computed tomography, HRCT）评价病变仅累及黏膜、黏膜下层，未累及软骨和外膜层，无淋巴结及远处转移；③肿瘤远端边缘在气管镜可视范围内；④患者无法耐受手术或不接受手术治疗；⑤患者在静脉麻醉结合高频/常频喷射通气条件下插入硬质镜，然后进行支气管镜介入治疗，术中单独或联合圈套、激光（Neodymium-dopted Yttrium Aluminium Garnet, Nd: YAG）、氩等离子体凝固（argon plasma coagulation, APC）、二氧化碳冷冻、光动力治疗（photodynamic therapy, PDT）等技术；⑥患者在治疗前和治疗后立即进行影像学评估；⑦支气管镜介入治疗后4周-6周要进行随访；⑧一旦发现支气管镜介入治疗2个周期后腔内肿瘤仍残存，则实施挽救性外科手术。

### 1.2 研究方法

#### 1.2.1 近期疗效观察

治疗前后对患者呼吸困难分级评分、支气管管腔狭窄程度、支气管内径进行评价。呼吸困难分级评分用（英国医学研究委员会U.K. Medical Research Council）进行统计（0分-4分）。

#### 1.2.2 远期疗效观察

支气管镜介入治疗2个周期后进行评价。疗效包括完全缓解（complete remission, CR）、疾病稳定（stable disease, SD）、疾病进展（progressive disease, PD）^[6]^。

### 1.3 知情同意

所有入组患者均填写知情同意书。在支气管镜介入治疗前，临床医生关于患者治疗策略进行术前病例讨论，达成统一治疗意见。

### 1.4 统计学方法

采用SPSS 21.0进行统计学分析，计量资料采用均数±标准差（Mean±SD）表示，计数资料采用百分比表示，计量资料的比较采用*Wilcoxon*秩和检验，计数资料的比较采用卡方检验，*P* < 0.05为有统计学差异。

## 2 结果

### 2.1 一般资料

经筛选共纳入32例患者（男性14例和女性18例），中位年龄44.5岁，从23岁-77岁。男性患者（14例）吸烟者10例（男性吸烟率71%，95%CI：30%-95%）；32例患者咯血14例（发生率44%，95%CI：21%-70%），伴阻塞性肺炎18例（发生率56%，95%CI：31%-79%）；术前支气管动脉栓塞术18例（栓塞率56%，95%CI：31%-79%）；介入治疗方式包括PDT15例，电圈套31例，冷冻32例，APC21例，Nd: YAG激光15例，光动力治疗15例，见表 1。

**表 1:** 患者一般状况、肿瘤特征、治疗策略和随访 Patient and tumor characteristics, treatment strategy and follow-up

No, male, age (years)	Smoking history	Hemoptysis	With obstructive pneumonia	Location	Bronchial artery embolization	Dimension (mm)	The distal margin of the tumor in the vision	BT	ICH	CR	Lobectomy	Follow-up（mon）
1, F, 33	No	Yes	No	LUL	Yes	5	Yes	1×electric snare/1×freeze/1×APC/3×PDT	CgA(+), NSE(+), S-100(-), CEA(-), EMA(+), TTF-1(+)	Yes	No	63
2, F, 36	No	No	Yes	RMB	Yes	13	Yes	2×electric /2×freeze/snare/2×APC	NSE(+), Cg-A(+), S-100(-)	Yes	No	59
3, M, 43	Yes	Yes	No	LUL	Yes	5	Yes	1×electric snare/1×freeze/1×APC/3×PDT	NSE(+), S-100(+)	Yes	No	28
4, M, 29	No	No	Yes	LLL	Yes	12	Yes	1×electric snare/1×freeze/1×APC	EMA(+), TTF-1(+), NSE(+),	Yes	No	38
5, M, 28	Yes	No	No	RB6	No	10	No	2×electric snare/2×freeze/2×APC	EMA(-), NSE(+)	No	Yes	28
6, F, 64	No	No	Yes	LMB	Yes	10	Yes	1×electric snare/1×freeze/1×APC/1×Nd: YAG laser/2×PDT	EMA(+), CgA(+), NSE(+), S-100(-)	Yes	No	23
7, F, 77	No	No	No	BI	No	13	Yes	1×electric snare/1×freeze/1×APC/2×PDT	NSE(+), CgA(-), S-100(-)	Yes	No	22
8, M, 33	Yes	Yes	Yes	LMB	No	10	No	2×electric snare/2×freeze/2×APC	CgA(+), NSE(+), S-100(+)	No	Yes	25
9, F, 23	No	Yes	Yes	RMB	No	12	No	2×electric snare/2×freeze/2×APC /2×Nd: YAG laser	NSE(+), S-100(-), CgA(+)	No	Yes	20
10, F, 68	No	No	No	RUL	Yes	7	Yes	1×electric snare//1×freeze/1×APC/3×PDT	CgA(+), NSE(-), S-100(-)	Yes	No	22
11, M, 51	No	No	No	RB6	No	10	No	2×electric snare/2×freeze/2×APC	CgA(+), NSE(+), S-100(-)	No	Yes	11
12, F, 57	No	No	No	BI	Yes	5	Yes	1×electric snare/1×freeze/1×Nd: YAG laser/3×PDT	CgA(+), NSE(+), S-100(-)	Yes	No	14
13, F, 32	No	Yes	Yes	RMB	Yes	7	Yes	1×electric snare/1×freeze/1×Nd: YAG laser/3×PDT	CgA(+), NSE(+)	Yes	No	6
14, F, 57	No	No	Yes	RMB	No	13	Yes	1×electric snare/1×freeze/1×Nd: YAG laser/3×PDT	NSE(+), S-100(+), TTF-1(+)	Yes	No	11
15, M, 57	Yes	Yes	Yes	RMB	No	12	Yes	1×electric snare/1×freeze/1×Nd: YAG laser/3×PDT	NSE(+), S-100(+), TTF-1(+)	Yes	No	6
16, F, 46	No	Yes	Yes	RMB-BI	Yes	15	Yes	1×electric snare/1×freeze/1×Nd: YAG laser/3×PDT	NSE(+), CgA(-), CD34(+)	Yes	No	4
17, M, 46	Yes	No	Yes	LLL	No	12	Yes	1×electric snare/1×freeze/1×APC	CgA(+), NES(+), CEA(-)	Yes	No	59
18, M, 68	Yes	Yes	Yes	RUL	No	6	Yes	1×electric snare/1×freeze/1×APC	NSE(+), Cg-A(+), S-100(-)	Yes	No	15
19, M, 74	No	Yes	No	BI	Yes	9	Yes	1×electric snare/1×freeze/1×APC/3×PDT	NSE(+), Cg-A(+), S-100(-)	Yes	No	72
20, M, 28	No	No	Yes	BI	Yes	14	Yes	1×electric snare/1×freeze/1×APC/3×PDT	NSE(+), S-100(+), TTF-1(+)	Yes	No	60
21, M, 65	Yes	Yes	Yes	LUL	Yes	16	Yes	1×Nd: YAG laser/1×freeze/1×APC	NSE(+), S-100(+)	No	Yes	3
22, M, 49	No	No	No	LUL	No	16	Yes	1×electric snare/1×freeze/1×APC	NSE(+), S-100(+)	No	Yes	48
23, F, 64	Yes	Yes	No	LMB	Yes	12	Yes	1×electric snare/1×freeze/1×APC/3×PDT	NSE(+), Cg-A(+), S-100(-)	Yes	No	26
24, F, 66	No	No	No	RMB	No	8	No	1×electric snare/1×freeze/1×APC/1×Nd: YAG laser	NSE(+), S-100(+), TTF-1(+)	No	Yes	54
25, M, 74	Yes	Yes	Yes	BI	Yes	14	No	1×electric snare/1×freeze/1×APC	NSE(+), S-100(+)	No	Yes	51
26, F, 51	No	No	Yes	RB6	Yes	10	Yes	1×electric snare/1×freeze/1×Nd: YAG laser	NSE(+), S-100(+), TTF-1(+)	Yes	No	20
27, F, 46	No	Yes	No	RMB	No	10	Yes	1×electric snare/1×freeze/1×Nd: YAG laser	NSE(+), S-100(+), TTF-1(+)	Yes	No	22
28, F, 58	No	No	Yes	RMB	No	8	Yes	1×electric snare/1×freeze/1×Nd: YAG laser	NSE(+), S-100(+)	Yes	No	16
29, F, 49	No	Yes	No	LMB	Yes	7	Yes	1×electric snare/1×freeze/1×Nd: YAG laser/1×APC/2×PDT	NSE(+), S-100(+), TTF-1(+)	Yes	No	25
30, F, 61	No	No	No	RB6	Yes	12	Yes	1×electric snare/1×freeze/1×Nd: YAG laser	NSE(+), S-100(+), TTF-1(+)	Yes	No	22
31, F, 58	No	No	Yes	RMB	Yes	10	Yes	1×electric snare/ 1×Nd: YAG laser	NSE(+), Cg-A(+), S-100(-)	Yes	No	18
32, M, 43	Yes	No	Yes	BI	No	12	Yes	1×electric snare/1×APC/3×PDT	NSE(+), S-100(+), TTF-1(+)	Yes	No	24
COPD: chronic obstructive pulmonary disease; LMB: left main bronchus; LLL: left lower lobe; RB6: right bronchial segment number 6; RMB: right main bronchus; BI: bronchus intermedius; LUL: left upper lobe; RUL: right upper lobe; RLL: right lower lobe; BT: bronchoscopic treatment; Nd: YAG laser: Neodymium-dopted Yttrium Aluminium Garnet; PDT: photodynamic therapy; CR: complete remission; NSE: neuron specific enolase; Cg-A: chromogranin A; TTF-1: thyroid transcription factor 1; EMA: epithelial membrane antigen; CEA: carcinoma embryonic antigen; F: female; M: male.												

### 2.2 气管镜下特征及患者随访

治疗前支气管镜下特征见表 1，包括腔内肿瘤部位、肿瘤大小、镜下是否可见肿瘤远端边缘等。使用支气管镜介入治疗具体技术和治疗结果见表 1。支气管镜介入治疗未发生并发症，未发生严重腔内出血。患者随访见表 1。部分患者支气管镜介入治疗前后胸部CT和支气管镜介入对比见图 1。

**图 1:**
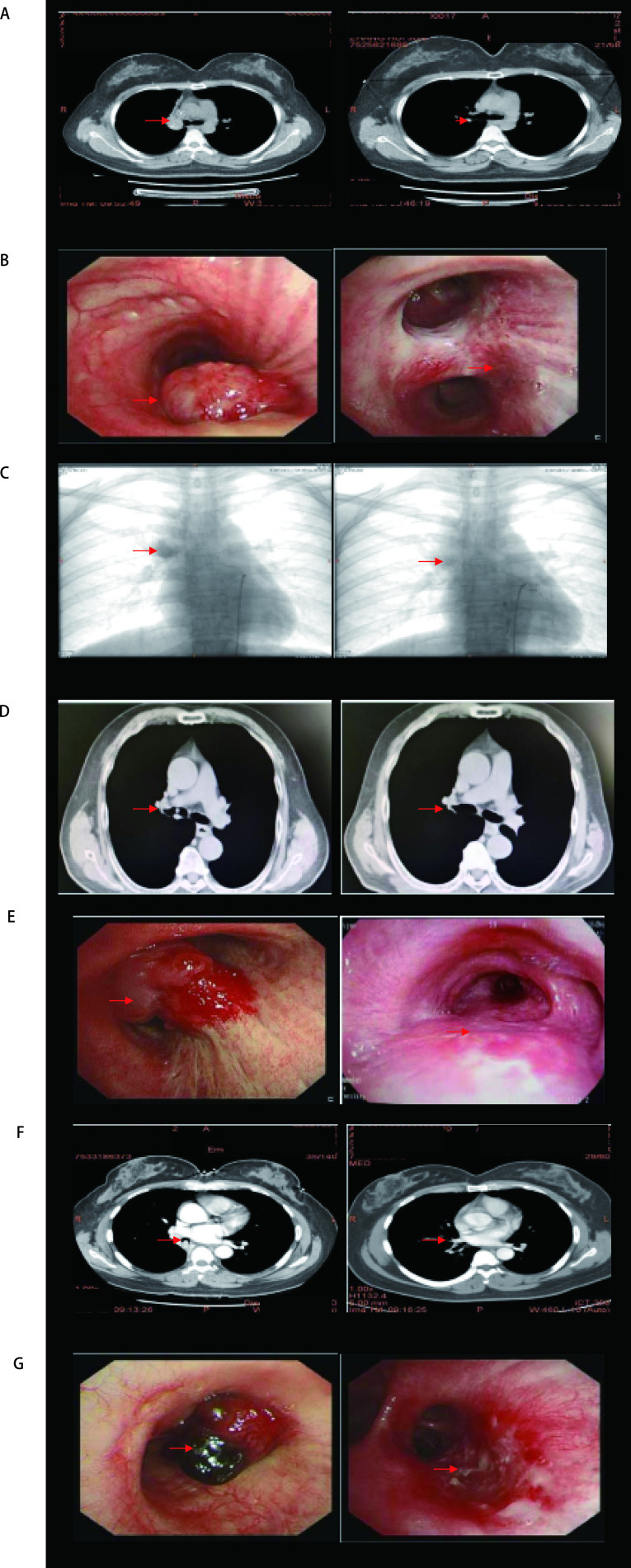
（A、B、C）对比图：A：病例13支气管镜介入治疗前后胸部CT对比；B：病例13支气管镜介入治疗前后对比；C：病例13支气管介入栓塞术前后对比；（D、E）对比图：D：病例14支气管镜介入治疗前后胸部CT对比；E：病例14支气管镜介入治疗前后对比；（F、G）对比图：F：病例16支气管镜介入治疗前后胸部CT对比；G：病例16支气管镜介入治疗。 Comparison chart (A, B, C). A: Comparison of thoracic CT scan in the before and after the bronchoscopic treatment for case 13; B: Comparison of bronchoscopy in the before and after the bronchoscopic treatment for case 13; C: Comparison of image outcome in the before and after the chemotherapy with bronchial arterial embolization for case 13. Comparison chart (D, E): D: Compari-son of thoracic CT scan in the before and after the broncho-scopic treatment for case 14; E: Comparison of bronchoscopy in the before and after the bronchoscopic treatment for case 14. Comparison chart (F, G): F: Comparison of thoracic CT scan in the before and after the bronchoscopic treatment for case 16; G: Comparison of bronchoscopy in the before and after the bronchoscopic treatment for case 16. CT: computed tomography.

### 2.3 近期疗效

呼吸困难分级评分治疗后较治疗前下降，差异有统计学意义[（1.44±1.03）分*vs*（0.25±0.58）分，*P*=0.003]；支气管管腔狭窄程度治疗后差较治疗前下降，差异有统计学意义[(87.50%±13.90%) *vs* (17.50%±6.83%), *P* < 0.001]；支气管内径治疗前与治疗后相比扩宽，差异有统计学意义[(0.14±0.18) cm *vs* (0.84±0.29) cm，*P* < 0.001]。近期有效率为100%。见表 2。

**表 2:** 支气管镜介入治疗前后指标变化 Comparison of relative indicator before and after the bronchoscopic treatment

	Before the treatment	After the treatment	*P*
Dyspnea grading score	1.44±1.03	0.25±0.58	0.003
The degree of stenosis (%)	87.50±13.90	17.50±6.83	< 0.001
Lumen diameter (cm)	0.14±0.18	0.84±0.29	< 0.001

### 2.4 远期疗效

32例患者中24例支气管镜介入治疗能够完全消瘤，CR率为75%（95%CI: 47%-92%）。在随访期间，间隔3个月-4个月复查支气管镜。支气管镜介入治疗后中位随访时间为22.5个月（范围：34个月-72个月）。光动力治疗15例患者，中位随访时间23个月（范围：4个月-72个月），其中3例患者达到根治，见图 1、图2、图3。在其他8例患者随之实施根治性外科手术，中位随访时间为26.5个月（范围：3个月-54个月）。如果患者进行根治性外科手术，术后无需进行支气管镜检查。

## 3 讨论

众所周知，来源于肺部的神经内分泌肿瘤包括典型类癌、非典型类癌与小细胞肺癌及大细胞神经内分泌癌。支气管肺类癌可发生于任何年龄，发病高峰年龄在50岁-56岁^[7]^，本研究中位年龄44.5岁。类癌患者的临床表现取决于肿瘤的大小、位置及生长方式。肿瘤组织富血供，患者会有咯血、呼吸困难、咳嗽、气道阻塞、发热、胸痛等临床表现，其中该研究咯血发生率为44%。因肿瘤血供丰富，支气管介入治疗存在大出血风险为减少血供控制肿瘤生长，故术前推荐行支气管动脉栓塞术，本研究32例类癌栓塞率为56%，栓塞后镜下治疗时均出血较少，提高了手术效率，减少了术中大出血风险；气道阻塞会造成反复同一部位的肺炎、肺不张，其中本研究中阻塞性肺炎发生率为56%，应该值得临床医生重视。

本研究提示，典型类癌在经支气管镜下电圈套、APC、冷冻等治疗后再结合光动力治疗，能够达到完全消瘤的效果。气管支气管恶性肿瘤患者可能存在气道阻塞症状，支气管镜介入治疗不仅可帮助减轻气道阻塞症状，而且可完全消除腔内肿瘤，为腔内多发、隐匿性肿瘤提供了治愈可能性^[8]^。本研究发现近期疗效显著，支气管镜介入治疗后呼吸困难分级评分和支气管狭窄程度明显改善，支气管内径增宽，提示介入治疗对于近期扩宽气道，缓解呼吸困难有重要作用。本研究中针对一个病灶使用多种技术联合，包括电圈套、APC、冷冻、Nd: YAG激光、PDT等，根据病灶特点，医师选择个体化治疗原则。对于腔内型、管壁型及部分混合型气道类癌，在削瘤后可以选择光动力疗法治疗。光动力疗法是将光敏剂血卟啉注入体内，肿瘤组织选择性摄取光敏剂，48 h后用特定波长激光局部照射后，产生光化学效应，使管腔内残存的肿瘤组织进一步坏死脱落，部分类癌可达到根治的效果。因此，PDT可能成为最大程度保留正常肺功能的肿瘤治疗方案^[9]^。然而，部分患者会出现光敏反应，由于避光时间比较长，可能为患者带来生活上的不便。

在我们的入组病例中，肿瘤位于支气管腔内，且肿瘤直径中位数为10 mm（范围：5 mm-15 mm）。HRCT提示肿瘤未侵犯支气管周围组织，我们通过联合圈套、ND: YAG激光、APC、二氧化碳冷冻先清除腔内肿瘤，之后再对残留肿瘤的基底部分给予PDT，使得肿瘤进一步脱落坏死，坏死深度达5 mm-10 mm，5例经PDT治疗的患者达到治愈，避免外科手术之苦。某些典型类癌肿瘤位置特殊，外科手术难以切除，或者手术会切除相对较多的肺组织，对患者造成比较大的影响。然而，支气管镜介入治疗则可以保留尽量多的正常肺组织。

有文献^[10, 11]^报道，支气管镜介入治疗进行腔内消瘤，患者可获得很好的远期生存。TC与AC生物学行为有所不同，AC即使有淋巴结转移，根治性手术切除仍可获得很好的远期存活。如果TC远端延伸至段支气管以下，支气管镜往往看不见肿瘤远端边缘，故在治疗难度上更大。例如病例5和病例11，肿瘤位于右下肺背段支气管，在支气管镜介入治疗过程中和治疗后，肿瘤远端是否侵犯均很难进行评估。因此，支气管镜介入治疗不能完整消除肿瘤，支气管镜介入治疗后肿瘤复发，最后还是进行挽救性手术。

尽管本研究发现支气管镜介入治疗典型类癌近期疗效和远期疗效显著，但是本研究的局限性亦不容忽视。首先，样本量少，本研究的样本量为32例，结果不一定客观准确。其次，本研究是回顾性分析，且患者的介入治疗技术多样，其结果有待大规模的随机对照临床研究进一步验证。

综上所述，对于低度恶性的TC而言，支气管镜介入治疗可能是一种有效的初始治疗，但是，支气管镜介入治疗在临床中的使用策略有待进一步研究，如各种技术如何优化搭配，疗效更显著，PDT使用周期数，能量密度如何选择等问题。此外也期待着更多的研究探讨支气管镜介入治疗技术的疗效及安全性，并进一步完善类癌患者的全程管理。
